# The hidden burden of dysmenorrhea among adolescent girls in Palestine refugee camps: a focus on well-being and academic performance

**DOI:** 10.1186/s12889-024-18219-0

**Published:** 2024-03-06

**Authors:** Rula Ghandour, Weeam Hammoudeh, Hein Stigum, Rita Giacaman, Heidi Fjeld, Gerd Holmboe-Ottesen

**Affiliations:** 1 Institute of Community and Public Health, Birzeit University, Occupied Palestinian territory (oPt) Said Khoury Building for Development Studies, Birzeit, P.O. Box 14, Palestine; 2 Department of Community Medicine and Global Health, University of Oslo, Postboks 1130 Blindern, 0318 Oslo, Norway

**Keywords:** Adolescent girls, Well-being, Dysmenorrhea, Academic performance, Palestinian refugee camps, West Bank and Jordan

## Abstract

**Background:**

Dysmenorrhea (painful menstruation) is a condition that may have a profound effect on adolescent girls’ health status and well-being. It can impede their engagement in daily activities and hamper their regular school attendance. This study aims to explore the relationship between dysmenorrhea, well-being, and academic performance among adolescent girls living in Palestine refugee camps in the West Bank and Jordan.

**Methods:**

We conducted a household survey between June and September 2019, with a total sample of 2737 adolescent girls 15 to 18 years old. Dysmenorrhea severity was assessed using the Working Ability, Location, Intensity, Duration of pain Dysmenorrhea scale (WaLIDD). The WHO-5 scale was used to evaluate the girls’ overall well-being. Menstrual academic disruption (MAD) was measured using a self-reported scale. Multiple linear regression models were employed to evaluate the association between dysmenorrhea, well-being, and academic performance. Directed Acyclic Graphs (DAGs) were employed to identify variables for control in regression models.

**Results:**

The mean dysmenorrhea score was 6.6 ± 2.6, with 37.9% and 41.2% expressing moderate and severe symptoms, respectively. The mean WHO-5 score was 58.7 ± 25.1, and 34.9% reported a low well-being status. The mean MAD score was 3.1 ± 3.3. 26% reported missing school due to dysmenorrhea, 36% said dysmenorrhea impacted their ability to concentrate, and 39% were unable to study for tests, and complete homework. The first regression analysis showed a reduction of 1.45 units in WHO-5 score for each unit increase in dysmenorrhea. The second regression analysis showed a non-linear increase in MAD score for increasing dysmenorrhea. For each dysmenorrhea score less than 4 (mild) there was a modest increase in MAD scores (coefficient 0.08, p-value = 0.006), and for each dysmenorrhea score above 4 there was a stronger increase in MAD scores (coefficient 0.95, *p* < 0.001).

**Conclusion:**

Dysmenorrhea poses significant challenges to the well-being and academic performance of adolescent girls living in Palestine refugee camps. Collaborative efforts and multifaceted approaches are crucial to address dysmenorrhea effectively. This involves research, targeted interventions, culturally sensitive strategies, and fostering a supportive environment that empowers girls to thrive academically and beyond.

**Supplementary Information:**

The online version contains supplementary material available at 10.1186/s12889-024-18219-0.

## Introduction

Dysmenorrhea, or menstrual pain, is painful cramps of uterine origin that happens during or before menstruation. The pain is usually located in the lower abdomen and sometimes in the back and thighs. It can be accompanied by systemic symptoms such as nausea, vomiting, headache, anxiety, and others, and can continue from 1 to 3 days [1, 2]. Dysmenorrhea is a common gynecological condition that affects women and girls worldwide [3, 4]. The most common form of dysmenorrhea is primary, occurring without underlying conditions and accounting for approximately 90% of cases among adolescents [5]. Primary dysmenorrhea can be relieved by both pharmacological therapy (such as non-steroidal anti-inflammatory drugs (NSAIDs)) or non-pharmacological therapy such as herbal remedies, heating, or regular exercise [6, 7]. Dysmenorrhea can also be secondary, signaling an underlying disease or condition such as endometriosis or pelvic inflammatory disease where medical attention is required [1, 6]. The global prevalence of dysmenorrhea ranges between 16 and 91% among women of reproductive age, with 2–29% reporting severe symptoms [5, 8, 9].

The main symptom of dysmenorrhea is pain which can be well understood within a bio-psycho-social pain model. Such a model recognizes the multifaceted nature of pain and considers the interconnected effects of biological, psychological, and social factors of pain experience. It suggests that pain perception extends beyond physical sensations alone, encompassing emotional well-being, stress levels, and the degree of support received from social networks [10, 11].

The bio-psycho-social pain model offers a comprehensive framework to understand the intricate complexities associated with dysmenorrhea and its effect on girls and women. The biological diemsion of dysmenorrhea is rooted in the biological processes of the menstrual cycle. During menstruation, the uterus contracts to shed its lining, and these contractions can lead to physical pain sensation, and discomfort [4, 5]. This physical pain can be potentiated by hormonal imbalances, increased prostaglandin production, and inflammation [4, 8]. The psychological dimension of dysmenorrhea can be manifested through negative emotions such as anxiety, frustration, and irritability due to pain and discomfort [12]. Dysmenorrhea is also influenced by social factors such as attitudes, socio-cultural norms, and social support systems that can shape how women perceive and manage this painful condition [13]. Furthermore, the social stigma surrounding menstruation may contribute to feelings of shame or embarrassment, impacting the individuals’ willingness to seek help or openly discuss their experiences [3, 4].

Dysmenorrhea by these different bio-psycho-social dimensions can have significant implications on adolescent girls’ health. The pain and discomfort associated with dysmenorrhea can have debilitating effects on their physical, emotional, and social well-being including aspects such as overall health, emotional resilience, and social interactions [4, 11]. This may in turn interfere with daily activities including schooling and social engagements, as well as contribute to feelings of isolation, anxiety, depressive symptoms, and reduced overall quality of life [8, 13, 14]. Studies across various contexts report that dysmenorrhea interferes with the daily lives of 20–50% of women and girls [15–18] and around 20% of students were subject to school absenteeism, with a higher prevalence observed in Low-Middle-Income Countries (LMIC) (26%) compared to High-Income countries (HIC) (12%), and with more than 40% of students reporting a negative effect on their concentration while studying, and this effect was consistent across both LMICs and HICs [19].

Adolescence is a critical period of transition to adulthood including a plethora of biological, emotional, psychological, and social changes [20]. At this critical juncture, the interconnected dynamics of well-being and academic achievement play a pivotal role in shaping the future prospects of adolescents [21]. The well-being of adolescents can be defined as their overall state of physical, mental, and social health. It encompasses various aspects of their lives, including their physical development, emotional well-being, social relationships, and access to resources and opportunities. It also takes into account the factors that influence their well-being, such as their family environment, community support, education, and access to healthcare [22]. On the other hand, academic achievement serves as a key measure of cognitive development and educational success during adolecence, offering insights into problem-solving skills and overall capacity to meet educational expectations [23].

Beyond well-being and academic performance, it is imperative to consider specific challenges faced by certain adolescent groups, such as adolescent refugees who undergo a unique set of experiences, navigating not only the typical challenges of adolescence but also the complexities of displacement and adaptation where their environment becomes even more pronounced in the context of disrupted lives and cultural adjustments [24, 25]. Adoelscent Palestine refugee camp dwellers are one example on such groups [26]. Palestine refugee camps are long-lasting camps established after the Israeli occupation of historic Palestine in 1948 and the West Bank in 1967. Adolescent girls living in these camps today embody third or fourth-generation refugees [27]. Previous research showed that adolescents living in Palestine refugee camps suffer from what is known as collective trauma resulting from prolonged occupation with significantly poorer qualtity of life compared to their adolescent counterparts [28–30]. Furthermore, and over time, the Palestine refugee camps have become closer to urban slums due to crowding and poverty, and Palestinean refugee needs over time have been transformed from the need for home and shelter to the need for education, employment, and improved infrastructure [31]. In addition to these constraints, adolescent girls living in these refugee camps experience the consequences of patriarchy controlling their lives, in addition to high levels of gender inequalities emerging from socio-cultural norms and values dominant in these camps [24, 27].

In this paper, we aim to examine dysmenorrhea as a bio-psycho-social phenomenon and its association with general well-being and academic performance within a context of political instability, patriarchial communities, and high levels of distress in Palestine refugee camps. The overall aim of the study is to provide insights that can inform targeted interventions and support systems to address the multifaceted challenges of dysmenorrhea faced by adolescent girls in this context and contribute to a broader understanding of the complexities faced by these girls and identify avenues for enhancing their overall health and educational experiences.

## Methods

This paper is part of a larger study that focused on adolescent girls living in Palestine refugee camps in the West Bank of the occupied Palestinian territory (oPt) (internally displaced) and Jordan (externally displaced). The primary objective of the big study is to address their primary health needs, including nutrition, anemia, reproductive health, and mental health [32]. Previous publications from this study delved into menstrual preparation, patterns, dysmenorrhea prevalence, and associated factors [33, 34]. The current paper specifically examines the effect of dysmenorrhea on general well-being and academic performance. 

### Study design and sampling

This paper relies on a cross-sectional household study conducted between June and September 2019. A representative sample of 2737 adolescent girls living in Palestine refugee camps in the West Bank of the oPt and Jordan was obtained. The sample was a stratified random sample proportional to refugee camp size. (see supplement 1 for the sample size calculation). Eligibility criteria included never-married adolescent girls 15–18 years old, living in any Palestine refugee camp in the West Bank (*N* = 19) and Jordan *(N = 10)* at the time of the survey.

### Study tools and data collection

The survey tool included two questionnaires, one targeting the mother or the primary female caregiver in the household, and the other addressed all adolescent girls between 15 and 18 years living in the household. All respondents were interviewed individually in private. The questionnaire for the mothers included information on household characteristics and economic status, while the questionnaire for the girls included detailed information related to dysmenorrhea, well-being, academic disruption related to dysmenorrhea, and associated factors which will be explained in the study variables section. Both questionnaires were piloted before starting data collection.

Trained Arabic-speaking women field workers collected data by using a random walk method. In each refugee camp, a series of random points were identified. The recruitment of households began at one of the random points, then all households on the right side from this point were included until completion, then another random point was chosen until the calculated sample size per stratum (camp) was attained.

### Study variables

The main exposure of interest for this study was dysmenorrhea measured using the Dysmenorrhea Scale (WaLIDD) assessed by Working Ability, Location, Intensity, Days of pain [35]. This multidimensional scale relies on four dimensions, as indicated by its name. Each dimension has four values (0–4). The final score of the WaLIDD scale ranges from 0 to 12 and was re-coded as indicated by the author into 4 main dysmenorrhea levels (0-No, 1–3: mild, 4–7: moderate, 8–12:severe) [35].

We had two main outcome variables: “well-being” and “disruption of academic performance”, by menstrual pain. Well-being was measured using the standardized Arabic version of the WHO-5 well-being tool [36]. The tool consists of five questions asking about the general well-being status during the last two weeks. Each question has a score from 0 to 5 in a positive direction in terms of well-being. The score was calculated by summing up the five questions and then multiplying the results by 4 to get a score ranging from 0 to 100. The disruption of academic performance was measured by the “Menstrual Academic Disruption (MAD)” scale that was developed based on our data. The scale consisted of three questions: Do you usually miss school days because of dysmenorrhea? Does dysmenorrhea affect your ability to concentrate or focus? Does dysmenorrhea affect your ability to do homework or study for exams? The outcome for each question consisted of a 5-point Likert scale ranging from 0 to 4, i.e.: 0: Never to 4: Always. The scale was calculated by summing up the values of the 3 questions resulting in a score of 0 to 12.

Other independent variables included were: age, country of location of the camp (West Bank of the oPt or Jordan), and the refugee camp locality being close to urban, rural, or Bedouin community. The Standard of Living score index (STL) was calculated as a proxy for economic status [37]. To calculate the index, each household was asked about the availability of a variety of different amenities (24 items). The score was then computed using factor analysis in which the components were forced into a single factor, and scores were derived by the regression method. The scale was then modified so that scores ranged from 0 to 100. Girls were also asked about their dietary diversity using the Minimum Dietary Diversity for Women of reproductive age (MDD-W) as a marker for food adequacy [38]. This included questions about the consumption of 10 different food items during the previous 24 h. Girls who reported less than five different food items per day were considered to have inadequate dietary diversity. Girls were also asked about some health behaviors, including skipping breakfast and being physically active. Menstrual health characteristics were also assessed, including age at menarche, menstrual cycle duration (number of bleeding days), and heaviness of bleeding, where girls were asked if blood leaks through their clothes as a proxy.

### Data analysis

In our analysis, there were two main dependent variables: the WHO-5 well-being scale and the Menstrual Academic Disruption (MAD) scale, both being continuous variables. For descriptive analysis, we used percentages and means with standard deviations depending on the measurement level for each variable. For bivariate analysis, we looked at mean scores within independent variable categories and used t-test and one-way ANOVA to test for any statistically significant variation. Finally, we conducted two multiple linear regression analyses models to assess dysmenorrhea’s association with well-being and menstrual academic disruption independently. Statistical significance was assessed at a significance level of 0.05.

**Fig. 1:**
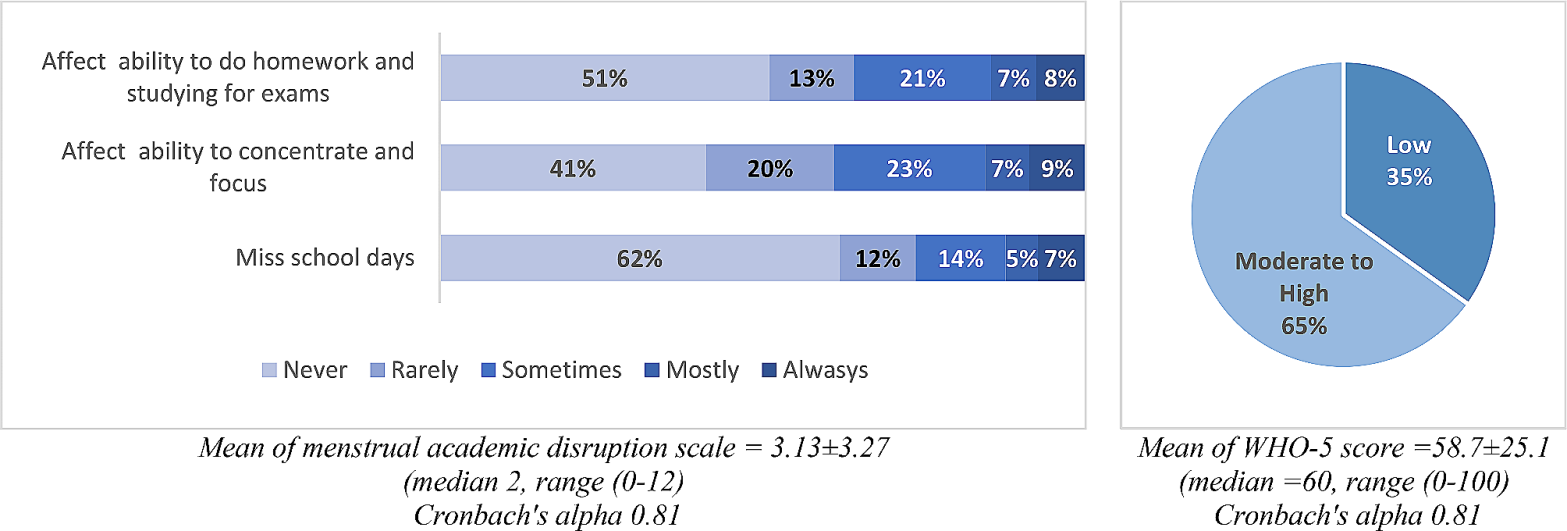
Menstrual academic disruption (MAD) scale items and WHO-5 well-being scale for adolescent girls living in Palestinian refugee camps in the West Bank and Jordan (*N* = 2657)

**Fig. 2:**
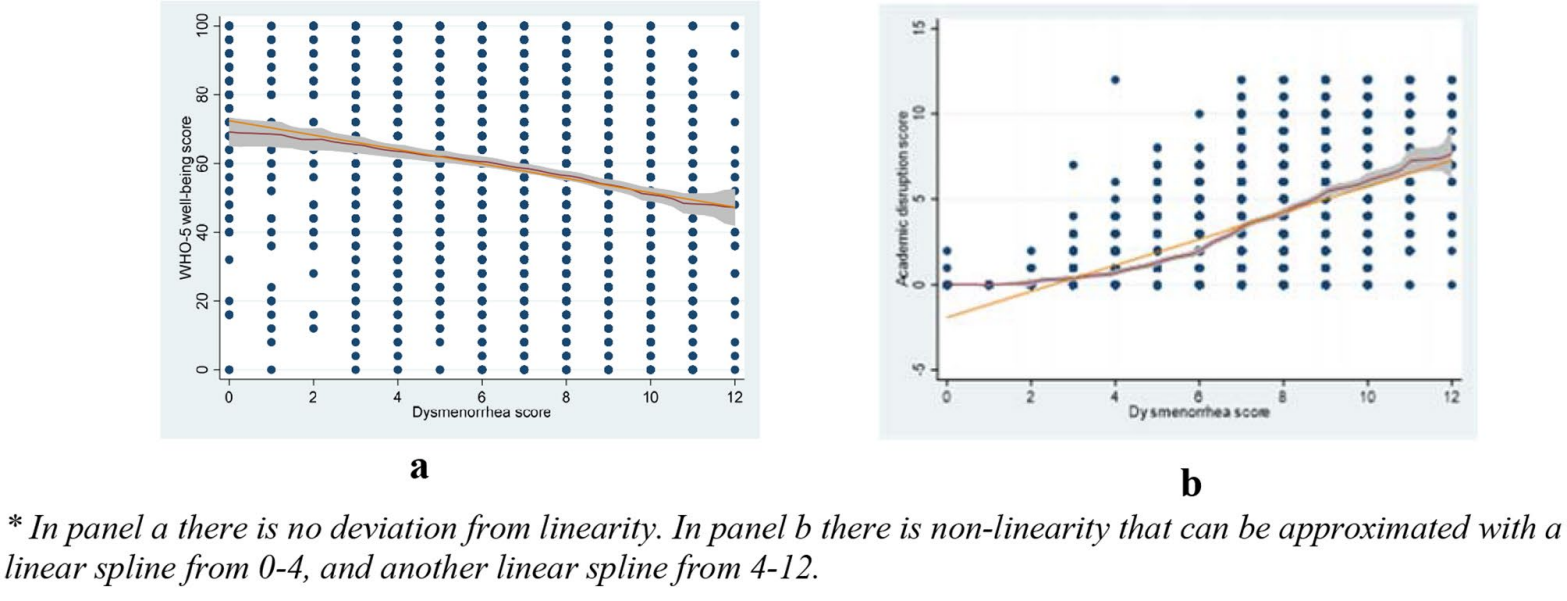
Plot of linearity in the association between dysmenorrhea score and well-being (WHO-5) (Panel a), and linearity in the association between dysmenorrhea score and menstrual academic disruption (MAD) scores (Panel b)*

We utilized the causal-directed acyclic graphs (DAGs) [39] to find out what to adjust/control for in the regression analysis for the two outcomes: WHO-5 and MAD. We visually presented the assumptions we tested through Directed Acyclic Graphs (DAGs) using the online software Dagity ® [40], see Supplement 2. We identified confounders, mediators, and colliders based on the literature and our knowledge of the relationship between exposures and outcomes. We controlled only for confounders as we aimed to assess the total effect of the exposure on the outcomes. Based on the DAGs for analysing the effect of dysmenorrhea on well-being, we identified confoudners as age, country, refugee camp locality, standards of living index, reported economic status, dietary diversity, skipping breakfast, physical activity, and age at menarche, and these were controlled for in the regression analysis (supplement 2-Fig. 1). Based on the DAGs for the effect of dysmenorrhea on academic performance, the same variables were controlled for except age at menarche (supplement 2-Fig. 2).

The assumptions for linear regression models were tested for both outcomes. Deviation from linearity was assessed by plotting the outcomes vs. the exposure and adding fit lines and fit curves. We used splines when there was a deviation from linearity in the association between the exposure and outcome. We also tested for constant error variance using the heteroscedasticity test and robust variance estimation. Because we included sisters in the study, we tested for independent residuals by comparing models with and without variance estimation with clustering for sisters. We checked for interactions between the main exposure (dysmenorrhea score) and all other independent variables controlled for in each model. Finally, we tested for influential outliers using the deltabeta plot. We used the software Stata 17® [41] for the analysis.

### Ethical considerations

Birzeit University’s Research Ethics Committee (Ref. No. 171,114) in the oPt and the Regional Committee for Medical and Health Research Ethics (REC) in Norway (Ref. No. 2018/2206) both gave their approval to the study in December 2017 and June 2018, respectively. Written informed consent for participation was obtained from the girls and their female caregivers in the same household. The data was collected privately to protect the girls’ privacy. The data were made anonymous before the analysis began.

## Results

A total of 2737 girls were eligible for the study (see supplement 1 for sample flowchart). Data on the main exposure (dysmenorrhea) and outcomes (well-being (WHO-5)) and Menstrual Academic Disruption (MAD) were available for 2657 girls. The mean age for participating girls was 16.8 ± 1.1 years. Half of the girls lived in refugee camps in the West Bank and the other half lived in Jordan. 73% were living in refugee camps close to urban areas, 23.3% close to rural areas, and 3.7% close to the Bedouin communities. The mean standard of living score was 37.8 ± 16.8. Still, 36.8% reported their economic status compared to those around them to be good to very good, whereas 50.8% reported it as fair, and 12.4% reported it as bad to very bad.

Concerning healthy behaviors, the mean dietary diversity score (MDD-W) among the girls was 5.2 ± 1.8, of whom 36.8% had inadequate dietary diversity. More than one-third of girls (38.1%) reported consistently skipping breakfast. 33% reported being physically active daily for at least 1 h, while 60.1% reported being physically active for 1–5 days per week and 6.7% were not physically active at all.

The mean age at menarche was 13.1 ± 1.2 years. Girls reported a range of 1 to 15 days of bleeding days, with a mean of 5.3 ± 1.5. When asked if blood leaks through their clothing as a proxy for the heaviness of their bleeding, 6.2% of respondents said ‘always’ and 16.5% said ‘occasionally’. For dysmenorrhea, the mean WaLIDD score was 6.6 ± 2.6, and only 3.7% reported no dysmenorrhea (WALIDD score = 0). The prevalence of mild, moderate, and severe dysmenorrhea was 17.2%, 37.9%, and 41.2%, respectively. See Table 1 for sample characteristics.

**Table 1:** Sample characteristics of adolescent girls living in Palestine refugee camps in the West Bank and Jordan *N* = 2657

**Demographic and socioeconomic characteristics**		**N**	**%**
Age (years) Mean (16.8 ± 1.1)	15	851	32.0
	16	674	25.4
	17	668	25.1
	18	464	17.5
Country	West Bank	1,329	50.0
	Jordan	1,328	50.0
Refugee camp locality	Urban	1,939	73.0
	Rural	620	23.3
	Bedouin	98	3.7
Standards of living score Mean (37.8 ± 16.8)	Low STL 1–30	995	37.4
	Middle STL 31–60	1,348	50.7
	High Stl 61–100	314	11.8
Reported economic status compared to others	Bad to very bad	329	12.4
	Fair	1,350	50.8
	Good to very good	978	36.8
***Behavioral characteristics***			
Dietary adequacy MDD-W mean (5.2 ± 1.8)	Adequate (≥ 5 DDS)	1,678	63.2
	Inadequate (< 5 DDS)	979	36.8
Skipping breakfast	Never	790	29.8
	Rarely	537	20.3
	Sometimes	313	11.8
	Always	1009	38.1
Number of days of being physically active for more than an hour/day Mean (4.4 ± 2.1)	0 days	177	6.7
	1–5 days per week	1598	60.1
	6–7 days per week	882	33.2
Number of bleeding days Mean (5.3 ± 1.5)	Short < 3 days	40	1.5
	Regular 3–7 dyas	2496	94.2
	Long > 7 days	114	4.3
Heaviness of bleeding (Blood leaks through clothes)	Never	1627	61.3
	Rarely	423	16.0
	Sometimes	437	16.5
	Mostly / Always	165	6.2
Dysmenorrhea Mean (6.6 ± 2.6)	No (WaLIDD = 0)	98	3.7
	Mild (WaLIDD 1–4)	458	17.2
	Moderate (WaLIDD 5–7)	1006	37.9
	Severe (WaLIDD 8–12)	1095	41.2

The WHO-5 well-being scale also yielded good internal consistency, with Cronbach’s alpha = 0.81. The mean WHO-5 well-being score was 58.7 ± 25.1 (range 0-100), with 35% reporting low well-being status. The Menstrual Academic Disruption (MAD) scale yielded good internal consistency with Cronbach’s alpha = 0.81 for the three items. The mean MAD score was 3.13 ± 3.27 (range 0–12) with moderaltely skewed distribution and a median of 2. According to Figs. 1 and 36% of participants were found to have their capacity to complete homework and prepare for exams (8% always, 7% mostly, and 21% sometimes) affected by dysmenorrhea, 39% said that dysmenorrhea affects their ability to focus or concentration (9% always, 7% mostly and 23% sometimes), and 26% said that they miss school days as a result of the dysmenorrhea (7% always, 5% mostly and 14% sometimes).

Bivariate analysis showed that the WHO-5 well-being score decreased significantly by increasing dysmenorrhea levels, increasing age, living in Jordan compared to the West Bank, inadequate dietary diversity, and skipping breakfast. WHO-5 scores were significantly higher for girls living in camps close to rural areas compared to urban areas, with better standards of living and economic status, and with being physically active 6–7 days per week compared to not being physically active at all. Furthermore, the WHO-5 well-being score was lower for long menstrual cycles compared to regular cycles. Although the negative association between the WHO-5 score and heaviness of bleeding was detected, this was not statistically significant.

On the other hand, MAD scores increased significantly with higher levels of dysmenorrhea, older age, living in Jordan compared to the West Bank, inadequate dietary diversity, and skipping breakfast. MAD scores tended to decrease significantly with living in camps close to rural areas, with better economic status, and with being physically active 6–7 days per week compared to not being physically active at all. Furthermore, MAD scores were higher for girls with longer menstrual cycles and increased with the increasing heaviness of bleeding. See Table 2.

**Table 2:** Bivariate association between sample characteristics and wellbeing (WHO-5) and Menstrual academic disruption (MAD) among adolescent girls in Palestine refugee camps (*N* = 2657)

		Well-being (WHO-5) *N* = 2657 Mean ± SD = 58.7 ± 25.1				Menstrual academic disruption (MAD) *N* = 2657 Mean ± SD = 3.1 ± 3.3			
		N	Mean	Mean diff	P-value*	N	Mean	Mean diff	P-value*
Dysmenorrhea	No	98	71.4	*ref*		98	0.0	*ref*	
	Mild	458	65.0	-6.4	0.019	458	0.5	0.4	0.148
	Moderate	1006	60.5	-11.0	< 0.001	1,006	2.3	2.3	< 0.001
	Severe	1095	53.2	-18.2	< 0.001	1,095	5.3	5.2	< 0.001
Age (years)	15	851	61.5	*ref*		851	2.7	*ref*	
	16	674	59.1	-2.5	0.058	674	3.2	0.6	0.001
	17	668	56.4	-5.1	< 0.001	668	3.3	0.7	< 0.001
	18	464	56.0	-5.5	< 0.001	464	3.5	0.8	< 0.001
Country	West Bank	1329	59.5	*ref*		1,329	2.9	*ref*	
	Jordan	1328	57.8	-1.7	0.081	1,328	3.3	0.4	0.003
Type of camp community	Urban	1939	58.0	*ref*		1,939	3.3	*ref*	
	Rural	620	62.1	4.1	< 0.001	620	2.5	-0.8	< 0.001
	Bedouin	98	50.2	-7.7	0.003	98	3.4	0.1	0.873
Standard of living score	Low STL 1–30	995	53.5	*ref*		995	3.7	*ref*	
	Middle STL 31–60	1348	61.4	7.9	< 0.001	1,348	3.0	-0.5	0.001
	High STL 61–100	314	63.3	9.8	< 0.001	314	2.6	-0.9	< 0.001
Reported economic status	Bad to very bad	329	45.9	*ref*		329	3.7	*ref*	
	Fair	1350	57.6	11.7	< 0.001	1,350	3.7	-0.4	0.055
	Good to very good	978	64.4	18.4	< 0.001	978	3.3	-1.0	< 0.001
Dietary adequacy	Adequate	1678	60.0	*ref*		1,678	3.0	*ref*	
	Inadequate	979	56.3	-3.8	< 0.001	979	3.4	0.4	0.001
Skipping breakfast	Never	790	62.4	*ref*		790	2.7	*ref*	
	Rarely	537	60.4	-2.0	0.154	537	3.3	0.6	0.001
	Sometimes	313	62.0	-0.4	0.809	313	3.3	0.6	0.01
	Always	1009	53.8	-8.6	< 0.001	1,009	3.3	0.6	< 0.001
Number of days of being physically active for more than an hour/day	0 days	177	49.6	*ref*		177	3.6	*ref*	
	1–5 days per week	1598	56.4	6.8	0.001	1,598	3.3	-0.4	0.172
	6–7 days per week	882	64.6	15.0	< 0.001	882	2.7	-0.9	0.001
Number of bleeding days	Regular	2496	58.8	*ref*		2,496	3.1	*ref*	
	Short	40	62.2	3.4	0.399	40	3.6	0.5	0.348
	Long	114	53.5	-5.3	0.028	117	3.9	0.8	0.01
Heaviness of bleeding (Blood leaks through clothes)	No, never	1627	59.9	*ref*		1,627	2.7	*ref*	
	Yes, rarely	423	55.7	-4.2	0.002	423	3.2	0.5	0.002
	Yes, sometimes	437	57.4	-2.5	0.063	437	3.8	1.1	< 0.001
	Yes, most of the time or always	165	56.4	-3.5	0.088	165	5.0	2.3	< 0.001

* P-values based on t-tests and one-way ANOVA comparing each level of the covariate to the reference

In the multiple regression model for the association between dysmenorrhea and well-being, a negative linear effect was detected with a coefficient of -1.45; thus for each one-unit increase in dysmenorrhea, there was a 1.45 unit reduction in the well-being score (see Table 3: model 1b). No deviation from linearity was detected in the association between dysmenorrhea and well-being (see Fig. 2a). The test for heteroscedasticity indicated that there was non-constant error variance. However, when we used the robust variance estimation, there were no important differences in standard errors or p-values compared to the original model. In the final model, the residuals were found to be independent and no clustering in the effect of dysmenorrhea on well-being was detected between sisters. Furthermore, no interactions were detected between dysmenorrhea and other confounders on its effect on the well-being score, and no influential outliers were detected.

**Table 3:** Multiple linear regression models for the association between dysmenorrhea and well-being (Model 1), and the association between dysmenorrhea and menstrual academic disruption (MAD) (Model 2). (*N* = 2657)

**Well-being (WHO-5)**			**Coefficient**	**95%CI**	**p-value**
Model 1a: Crude model/unadjusted model		2567	-2.10	[-2.45,-1.74]	< 0.001
Model 1b: Adjusted model ^a^		2641	-1.45	[-1.82,-1.09]	< 0.001
**Menstrual academic disruption (MAD)**		**N**	**Coefficient**	**95%CI**	**p-value**
Model 2a: Crude model/ unadjusted model		2657	0.76	[0.73,0.80]	< 0.001
Model 2b: Adjusted model ^b^		2641	0.74	[0.70,0.78]	< 0.001
Model 2c: Adjusted model with linear splines ^c^	No to mild dysmenorrhea (score 0–4)	2641	0.07	[-0.05,0.19]	0.261
	Moderate and severe dysmenorrhea (score 4–12)		0.95	[0.90,1.00]	< 0.001
Model 2d: Adjusted model with linear splines and correction for clustering by sisters	No to mild dysmenorrhea (score 0–4)	2641	0.08	[0.02,0.13]	0.006
	Moderate and severe dysmenorrhea (score 4–12)		0.95	[0.90,1.00]	< 0.001

a: adjusted for age, country, locality of camp community, standards of living, reported economic status, skipping breakfast, dietary diversity, physical activity, menstrual cycle length, intensity of bleeding age at menarche

b: adjusted for age, country, locality of camp community, standards of living, reported economic status, skipping breakfast, dietary diversity, physical activity, menstrual cycle length, and intensity of bleeding

c: adjusted model with linear splines with at knot at dysmenorrhea = 4

In the adjusted multiple linear regression model, dysmenorrhea showed a significant positive association with academic disruption (MAD) (Table 3: model 2b). However, when checking model assumptions, we found a deviation from linearity in the association between dysmenorrhea and MAD, see Fig. 2b, thus we used linear splines in our analysis using the dysmenorrhea score = 4 as a knot point for the linear splines. (see Table 3: model 2c). Furthermore, we found the residuals to be dependent, thus, we reported the model after controlling for the clustering between sisters (see Table 3: model 2d), this also handles any heteroscedasticity. Finally, no interactions were detected between dysmenorrhea and other confounders, and no influential outliers were detected.

In the final multiple linear regression model, academic disruption increased significantly by 0.08 units for each one-unit increase for mild dysmenorrhea levels (WaLIDD score ≤ 4). However, this effect became much stronger when dysmenorrhea became moderate to severe (WaLIDD score > 4) with 0.95 units increase in academic disruption for each one unit increase in dysmenorrhea. See Table 3.

## Discussion

The findings of this study showed that high levels of dysmenorrhea had a debilitating effect on the lives of adolescent girls living in Palestine refugee camps in both the West Bank of the occupied Palestinian territory (oPt) and Jordan, significantly affecting their well-being and impeding their academic performance in several ways. These included increased school absenteeism, reduced ability to complete homework and prepare for exams, and reduced capacity to focus and concentrate in class.

The association between dysmenorrhea and well-being is supported by existing research, indicating that the pain and discomfort associated with dysmenorrhea can contribute to feeling down, frustrated, and/or unhappy, ultimately affecting the overall well-being and quality of life of girls [8, 18, 42, 43]. It has been reported that adolescent girls with dysmenorrhea have a higher risk of developing depressive symptoms and anxiety than those who do not suffer from dysmenorrhea [44]. In a study targeting university students in Turkey, dysmenorrhea was found to affect various dimensions of health-related quality of life (HRQL) measured by the SF36-short version. The findings revealed that the severity of dysmenorrhea was associated with the majority of the scale dimensions of HRQL [45]. Another study, targeting 12–18 years old adolescents showed that those who reported dysmenorrhea exhibited higher depression and anxiety scores and impaired quality of life compared to adolescents without dysmenorrhea. Furthermore, increased dysmenorrhea severity was associated with elevated depression and anxiety levels along with decreased psychosocial health subscale scores of quality of life [43]. These results are in line with our study that demonstrated a clear negative dose-response relationship between the severity of dysmenorrhea and well-being scores.

Moreover, many recent studies had addressed the effect of dysmenorrhea on academic performance, reflecting on the main dimensions we measured in our study: school absenteeism, ability to do homework, studying for exams, focusing, and concentrating in classes. In a recently published meta-analysis that encompassed data from both developed and developing countries, and included 19 studies and more than 11 thousand school and university students, researchers found that 18% of schoolgirls reported experiencing school absenteeism, while 45% reported impaired ability to focus, concentrate, or study for exams [19]. Comparable levels were reported in studies from the region including Jordan, Iraq, and Morrocco who reported school missing levels between 8 and 13% [46–48]. In Kuwait, studies reported even higher levels: 58% missed school due to pain, and 14% missed exams [49].

Our study aligns with the above mentioned findings. What sets our analysis apart is that we combined academic performance dimensions into a single scale called the Menstrual Academic Disruption (MAD) scale. This scale allowed us to observe how dysmenorrhea’s impact on academic performance varies with its intensity, creating a dose-response effect. Our results demonstrated that mild dysmenorrhea had a minimal effect on academic disruption, with a regression coefficient of 0.08. However, this coefficient increased significantly, around 12 times, when dysmenorrhea became moderate to severe. This indicates a strong relationship between the severity of dysmenorrhea and its negative influence on academic performance.

The effect of dysmenorrhea on well-being and academic performance appears to be evident through various dimensions within the bio-psycho-social dysmenorrhea pain model. The biological dimension of dysmenorrhea primarily encompasses physical symptoms such as pain, fatigue, nausea, and drowsiness. These symptoms often disrupt a girl’s ability to attend school and concentrate on academic tasks [8, 19]. The psychological dimension of dysmenorrhea can include anxiety, irritability, and mood swings that can further exacerbate pain perception and reduce pain tolerance [15]. Moreover, these psychological symptoms may also cause girls to be more self-conscious and ashamed of their bodies, and lower their self-esteem and motivation in participating in activities [43, 50]. It is important to highlight that the anxiety caused by dysmenorrhea may extend beyond menstruation days, impacting the overall well-being of girls throughout the month. This encompasses concerns about menstrual occurrence when setting up plans for studying or other activities [51]. From another perspective, the anxiety and irritability associated with dysmenorrhea can affect the relationships and communication of girls, potentially jeopardizing these relationships due to the manifestation of inappropriate behaviors [49].

The social dimension of dysmenorrhea also has a significant effectsthat can be observed in various ways. One way is through the cultural practice of treating menstruation with shame and taboo and by keeping menstruation a secret, which often leads to a lack of understanding and awareness regarding menstrual pain [34, 52, 53].This was clearly evident in earlier phases of this study [33, 54] and can potentially hinder students from seeking help or support [34, 55], particularly when dysmenorrhea is dismissed as a normal aspect of menstruation. Consequently, there may be underreporting and insufficient support for girls experiencing dysmenorrhea [3, 4, 17, 19]. This might be further compounded by limited access to healthcare services for adolescent girls, which can exacerbate the situation [55, 56].

Moreover, this internalization of stigma and taboo may lead to pain internalization, where girls’ experience of pain is incorporated in their internal thoughts, beliefs, and self-perception [52]. They may start to view themselves and their bodies in a negative way, which can lead to feelings of self-objectification, low self-esteem, and a tendency to isolate themselves from others during their menstrual period [57, 58].

Thus, dysmenorrhea presents a significant challenge for adolescent girls in general. However, we believe that it poses an even greater challenge for girls living in a refugee camp setting. This is particularly evident given the transformative phase that adolescent girls undergo after reaching menarche, marked by significant changes, and often compounded by limited available opportunities [59]. In the context of Palestine refugee camps, adolescent girls are constricted by their physical, social, and material spaces. The physical space is marked by overcrowding and inadequate infrastructure, obstructing their access to crucial resources and services. The social sphere operates as a mechanism of constraint, surveillance, and control, limiting girls’ autonomy by instilling a feeling of constant observation. Moreover, the material aspect is evidenced by challenging financial circumstances, further intensifying the hardships experienced by individuals already grappling with economic adversity [54, 60]. In this context, we can see dysmenorrhea with its high prevalence as a big obstacle to health and wellbeing for girls in refugee camps in the West Bank and Jordan.

The challenges faced by Palestinian refugees are not unique; similar situations are encountered by many displaced adolescents. An exploratory qualitative study on the sexual and reproductive health (SRH) perceptions and experiences of Syrian refugee adolescent girls in an urban setting in Lebanon revealed a substantial knowledge gap regarding menstruation, including dysmenorrhea, hindering effective coping mechanisms [61]. From another perspective, and directly linked to education and academic performance, it was reported that adoelecent refugee girls in Jordan face restrictive gender norms related to family honor, fears of sexual harassment on the way to school, and a lack of value placed on girls’ education. These factors, combined with the lack of understanding and support from teachers regarding girls’ needs during menstruation, can contribute to girls missing school during their menstrual cycles. Furthermore, it was reported that exams were rarely excused for girls during their menstrual cycles, and, unless in exceptional situations, they were not permitted to take a break or leave school to go home for relief from menstrual pains. This lack of support and understanding can have a negative impact on girls’ attendance and educational outcomes [62], especially that education is an important issue for the adolescent refugees and seen as a tool to overcome barriers [25]. In this discourse, as far as our knowledge extends, there have been no studies examining the impact of dysmenorrhea on academic performance and well-being among adolescent refugee camp dwellers.

Building on our analysis and its alignment with existing literature, it is evident that menstrual health, especially dysmenorrhea, should be given greater attention in public health interventions. Finding ways to help these girls to cope with menstrual pain and reduce the resulting stress might facilitate a better future for them in several ways. First, improved menstrual health education and access to resources create a foundation for informed decision-making and self-care practices, promoting long-term well-being [63]. By normalizing conversations around menstruation, reducing stigma, and fostering a supportive environment, girls may develop increased confidence and resilience [55]. Enhanced mental and emotional well-being, resulting from stress reduction initiatives and wellness programs, can positively impact academic performance and overall life satisfaction. This empowers girls to navigate their reproductive health with confidence, ensuring they can pursue education, career goals, and personal development unimpeded by the challenges of menstrual pain and stress [64–66].

Thus we advocate for bio-psycho-social assessment and management for dysmenorrhea. In school settings, this implies the need to help support students’ academic success by providing education and resources on menstrual pain management [19]. Schools can also consider implementing policies that allow for flexible attendance or accommodations for students who experience severe menstrual pain. Most importantly, addressing the social stigma surrounding menstruation and menstrual pain can also help create a supportive and inclusive school environment for all students [55].

Lastly, given the multi-dimentisional nature of dysmenorrhea, sometimes it needs to be assessed by a range of healthcare providers,, counselors, and psychologists [15, 43]. Underlying disease has to be ruled out, such as endometriosis - the most common cause of secondary dysmenorrhea [67] and would have to be diagnosed by medical personnel before adequate treatment is decided on. Proper training and preparation for these health professionals are crucially needed.

In general, managing dysmenorrhea involves a multifaceted approach that aims to minize menstrual pain and improve overall well-being. Lifestyle modifications, such as regular physical exercise and maintaining a balanced diet, have been associated with reduced menstrual discomfort. Additionally, dietary supplements like omega-3 fatty acids and vitamin B6 may offer relief. Non-pharmacological interventions, including heat therapy and acupuncture, have shown efficacy in alleviating pain [5, 68–70]. Furthermore, over-the-counter pain relievers, such as nonsteroidal anti-inflammatory drugs (NSAIDs), can provide effective symptomatic relief. A comprehensive and individualized strategy, considering both pharmacological and non-pharmacological options, can contribute to the successful management of dysmenorrhea [6].

### Strengths and limitations

The primary strength of this study lies in its large sample size, which enhances its representativeness of the study population and the generalizability of the study to the West Bank and Jordan’s adolescent girls populations living in refugee camps. Additionally, the detailed and in-depth analysis conducted provides a clear and multidimensional understanding of the situation. Furthermore, using directed acyclic graphs (DAGs) in identifying potential confounders, mediators, and colliders enhances the precision and robustness of the analyses. It is important to note, however, that we could not differentiate between primary and secondary dysmenorrhea in our study due to lack of resources. The cross-sectional design of this study imposes constraints on making causal inferences.

## Conclusion

In conclusion, dysmenorrhea, as a bio-psycho-social phenomenon, is a condition that can have serious implications and consequences on the lives of adolescent girls. Addressing dysmenorrhea requires a comprehensive approach that considers the broader biological, psychological, social, and cultural factors that contribute to its occurrence and impact. This includes increasing awareness and understanding of dysmenorrhea, promoting gender equality, and improving access to healthcare and support. We need to develop a thorough understanding of dysmenorrhea as a globally significant health issue that manifests in diverse ways throughout the lives of girls and women. This study serves as one of the foundational stepping stones to an improved understanding of dysmenorrhea and its effects on girls, paving the way for extensive future research to further expand our knowledge concerning this health issue. It is important for future research to prioritize the development of strategies aimed at enhancing the management of pain and symptoms to minimize the impact of dysmenorrhea. By targeting healthy habits and behaviors known to be effective in reducing dysmenorrhea levels, such as engaging in regular physical activity, consuming breakfast, and reducing smoking, young women can maximize their educational opportunities and improve their prospects for the future. By recognizing and addressing the challenges associated with dysmenorrhea, we can help women and girls to better manage symptoms and achieve greater physical, emotional, and social well-being.

### Electronic supplementary material

Below is the link to the electronic supplementary material.

Supplementary Material 1

Supplementary Material 2

## Data Availability

Data can be obtained through a suitable request; nevertheless, we retain the sole usage rights to the main results of the study until they are published. To access all available data, please get in touch with the corresponding author at rghandour@birzeit.edu.

## Abbreviations

WHO: World Health Organization
MAD: Menstruation academic disruption
PCBS: Palestinian Central Bureau of Statistics
oPt: occupied Palestinian territory
UNRWA: United Nations Relief and Works Agency in the Near East
WaLIDD: Working ability, Location, Intensity, Duration of pain Dysmenorrhea
