# Supplementary material for: The hidden burden of dysmenorrhea among adolescent girls in Palestine refugee camps: a focus on well-being and academic performance
Source: BMC Public Health. 2024 Mar 6;24:726. doi: 10.1186/s12889-024-18219-0 (PMC10918951; doi:10.1186/s12889-024-18219-0)
Supplement: Supplementary file 2 — Supplementary Material 2 [file 12889_2024_18219_MOESM2_ESM.docx]

**Supplement 2: Directed acyclic graphs (DAGs) for the effect of dysmenorrhea on academic performance (measured as menstrual academic disruption (MAD)) and general well-being**

*Note that several arrows between confounders have been left out. This simplifies the DAG, but will not influence the conclusions from the DAG.*

| 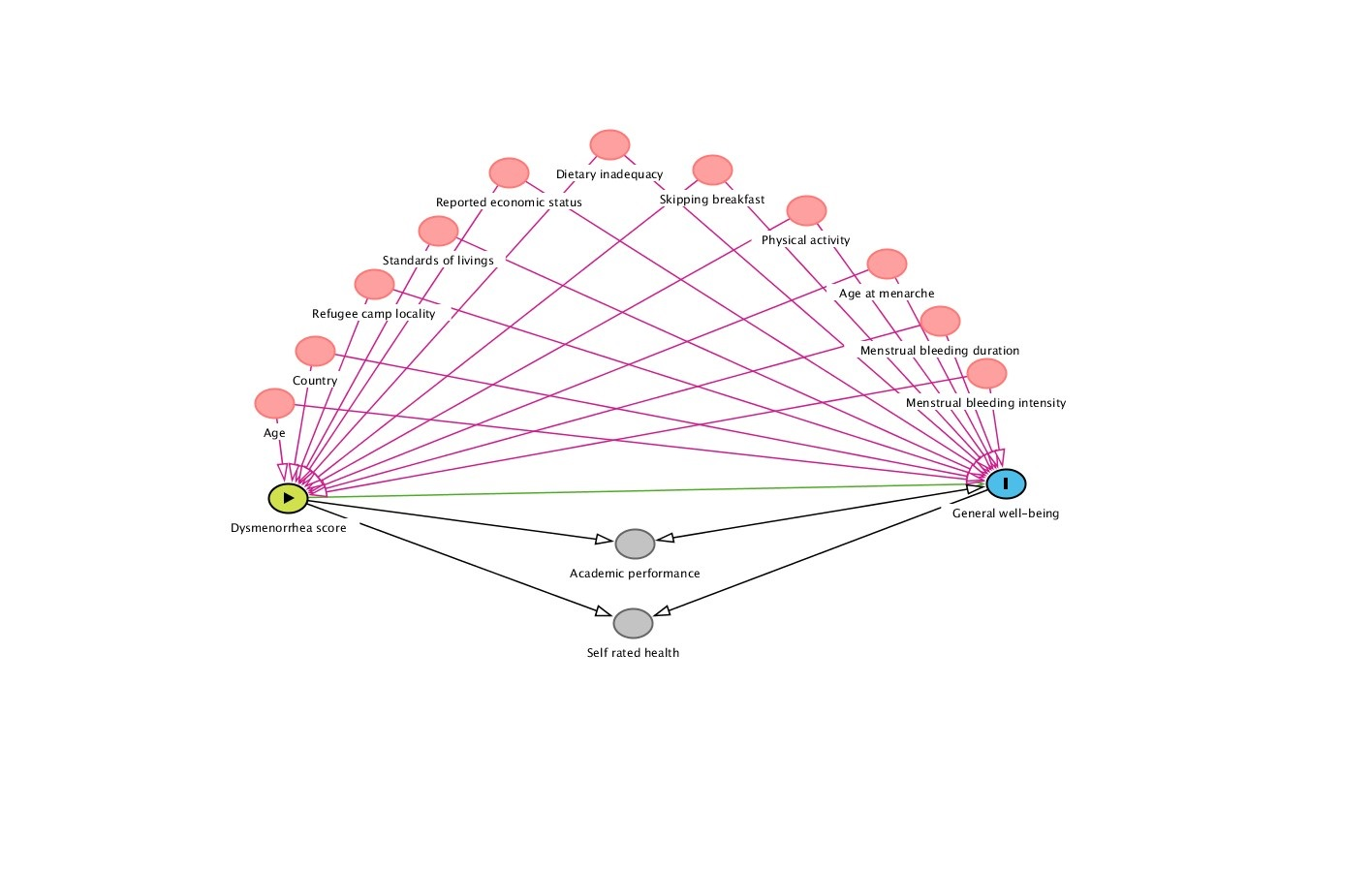 | | | | | |
| --- | --- | --- | --- | --- | --- |
| Figure 1 | | | | | |
| 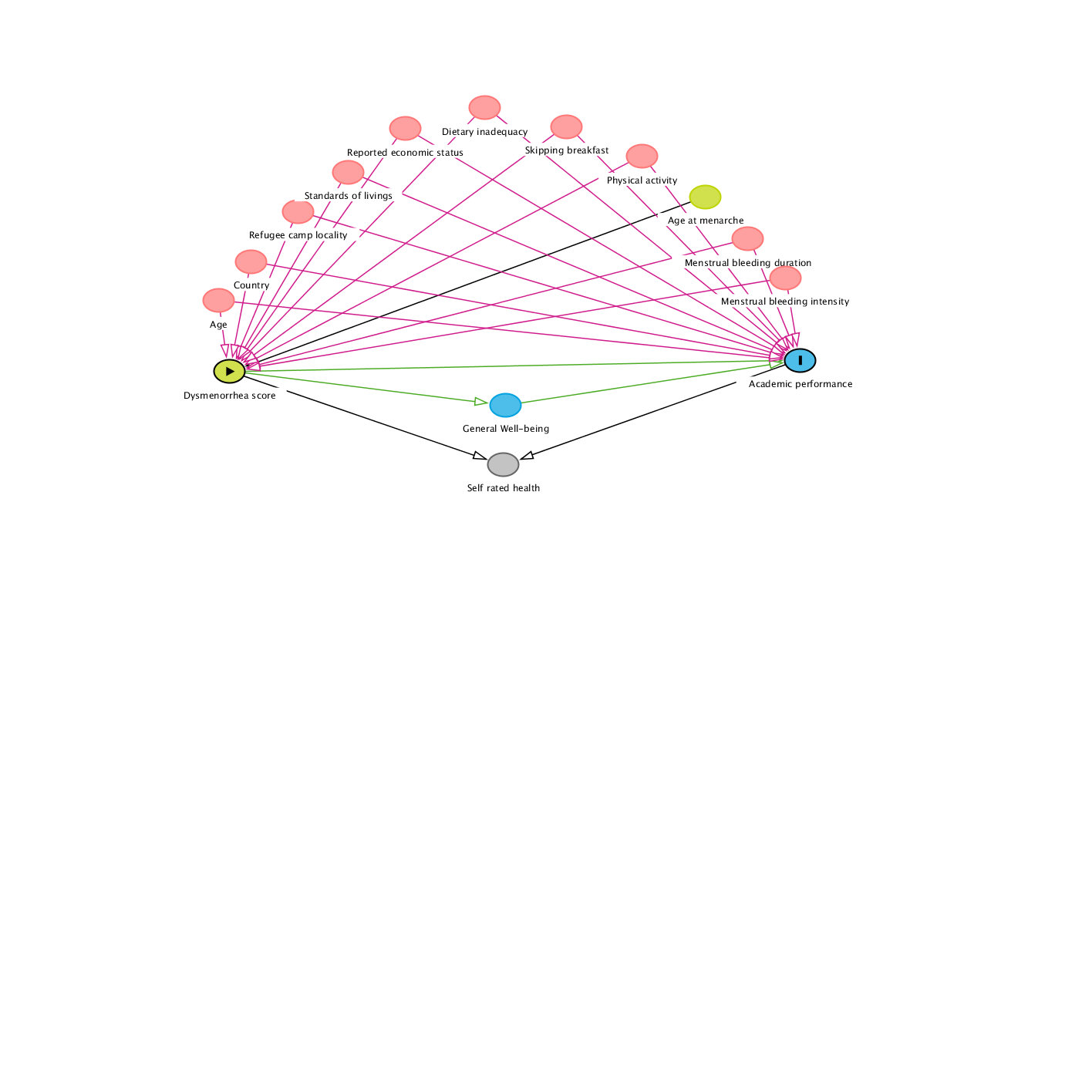 | | | | | |
| Figure 2 | | | | | |
| 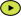 | Exposure | 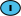 | outcome | 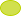 | Risk factor for the exposure |
| 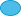 | Mediator | 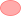 | Confounder | 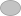 | Collider |
